# Identification of ferroptosis‐related genes as potential biomarkers of tongue squamous cell carcinoma using an integrated bioinformatics approach

**DOI:** 10.1002/2211-5463.13348

**Published:** 2021-12-24

**Authors:** Haisheng Zhu, Yuzhi Tao, Qingwen Huang, Zhuoming Chen, Liujun Jiang, Haolin Yan, Jinghua Zhong, Leifeng Liang

**Affiliations:** ^1^ Department of Oncology The Sixth Affiliated Hospital of Guangxi Medical University, The First People's Hospital of Yulin China; ^2^ Zunyi Medical University China; ^3^ Department of Respiratory and Critical Care Medicine Guizhou Provincial People's Hospital Guiyang China; ^4^ Department of Pathology The Sixth Affiliated Hospital of Guangxi Medical University, The First People's Hospital of Yulin China; ^5^ Department of Oncology The First Affiliated Hospital of Gannan Medical University Ganzhou China

**Keywords:** bioinformatics analysis, CA9, ferroptosis, prognosis, tongue squamous cell carcinoma

## Abstract

Tongue squamous cell carcinoma (TSCC) is one of the deadliest cancers of the head and neck, but the role of the ferroptosis pathway in its development is still unknown. In this study we explored the pathogenetic mechanisms associated with ferroptosis in TSCC. We identified differentially expressed genes (DEGs) of TSCC patients and used gene ontology (GO), the Kyoto Encyclopedia of Genes and Genomes (KEGG), and gene set enrichment analysis (GSEA) to annotate, visualize, and integrate these DEGs. Receiver operating characteristic curve (ROC) analysis was performed, and the STRING database was used to construct a protein–protein interaction network to evaluate the predictive value of ferroptosis‐related DEGs. A total of 219 DEGs were identified and GO, KEGG, and GSEA showed that extracellular matrix (ECM)‐receptor interaction and interleukin (IL)‐17 signaling pathways were substantially upregulated in TSCC. Univariate Cox analysis revealed that high expression of *CA9*, *TNFAIP3*, and *NRAS* were predictive of a worse outcome. We then constructed a prognostic model that predicted survival in the validation cohort at 1 year and 32 months. Finally, 60 cases of tongue carcinoma and normal tissues were collected, and immunohistochemistry was used to detect the expression of *CA9*. We found that *CA9* was strongly expressed in tongue carcinoma tissues and absent in adjacent tissues. Overall, we found that ferroptosis‐related genes may affect TSCC prognosis through the ECM‐receptor interaction and IL‐17 signaling pathways. Additionally, immunohistochemistry confirmed that *CA9* was highly expressed in tongue carcinoma tissues, and a model based on ferroptosis‐related genes showed a good ability to predict overall survival in TSCC.

## Graphical abstract

Here we identified differentially expressed genes (DEGs) in tongue squamous cell carcinoma (TSCC) patients and intersected them with ferroptosis‐related genes. Ferroptosis‐related genes may affect TSCC prognosis through the ECM‐receptor interaction and IL‐17 signaling pathways. We constructed a prognostic model based on a subset of the ferroptosis‐related genes and confirmed that one of these genes, *CA9*, is highly expressed in tongue carcinoma tissues.

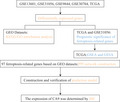

**Abbreviations**
AUC: area under the ROC curve
DEGs: differentially expressed genes
DLBCL: diffuse large B‐cell lymphoma
ECM: extracellular matrix
GEO: gene expression omnibus
GO: gene ontology
GSEA: gene set enrichment analysis
HR: hazard ratio
IHC: immunohistochemistry
IL‐17: interleukin‐17
KEGG: Kyoto Encyclopedia of Genes and Genomes
OS: overall survival
OSCC‐GB: oral squamous cell carcinoma of the gingivo‐buccal region
ROC: receiver operating characteristic
TCGA: The Cancer Genome Atlas
TSCC: tongue squamous cell carcinoma

Tongue squamous cell carcinoma (TSCC) accounts for 41% of all oral cancers; additionally, the tongue is rich in blood flow and metabolically active, making the metastasis rate higher than that in other types of oral squamous cell carcinoma [1]. Once metastasis begins, the 5‐year survival rate drops from 85.5% to 48.5% [2]. It has been shown that promoting ferroptosis and apoptosis can inhibit the growth and metastasis of TSCC cells [3]. However, the mechanism of ferroptosis and TSCC has not been studied. Therefore, to improve the therapeutic and diagnostic capabilities for TSCC, we investigated potential prognostic targets and mechanisms of ferroptosis.

Ferroptosis is a recently identified cell death pathway characterized by iron‐dependent accumulation of lipid hydroperoxides [4]. Studies have shown it to be associated with many diseases as a cell death mechanism different from autophagy or apoptosis. The authors of a study on diabetic rats found considerable iron‐dependent cell death in the hippocampus, confirming that ferroptosis is associated with diabetic cognitive impairment [5]. Chen et al. [6] found that promoting ferroptosis can inhibit the progression of osteosarcoma. Ferroptosis is closely related to drug resistance, sensitivity to chemotherapy, and metastasis of cancer cells [7], making it a crucial tumor inhibition mechanism with the potential to treat cancer. A study on tongue cancer showed that the tumor suppressor drug quisinostat could inhibit tumor cell growth by markedly inducing apoptosis and ferroptosis in TSCC cells [3]. Under pathological scenarios, ferroptosis often accompanies other cell death routines; however, inhibition of apoptosis or necroptosis is not sufficient to inhibit ferroptosis [8]. Therefore, modulating ferroptosis may have therapeutic potential in various ferroptosis‐associated diseases. Although ferroptosis is a critical factor in tumor prognosis, there is limited understanding of the role of ferroptosis in TSCC, especially the prognostic significance of ferroptosis‐related genes is still unclear.

At present, there are few studies related to ferroptosis in TSCC. Recent studies only propose the therapeutic effect of promoting the phenotype of ferroptosis without specific mechanisms and genes [9]. Although these studies demonstrate the importance of ferroptosis in TSCC, they do not examine the prognostic value of ferroptosis‐related genes for TSCC or differential expression in tumor tissues; therefore, previous studies lack a clear direction for further molecular research.

In this study we sought to identify potential diagnostic biomarkers and their biological functions associated with TSCC by mining the Gene Expression Omnibus (GEO), FerrDB (a website of genes related to ferroptosis), and The Cancer Genome Atlas (TCGA) databases. We used gene ontology (GO), Kyoto Encyclopedia of Genes and Genomes (KEGG), and gene set enrichment analysis (GSEA) to analyze biological processes involving the enriched genes to evaluate the diagnostic value of ferroptosis‐related genes in TSCC. The expression of CA9 was also verified by immunohistochemistry in tongue cancer samples. Our study provides new insights into potential regulatory mechanisms in TSCC patients.

## Materials and methods

### Patients and TSCC samples collection

Samples were collected for paired tumor and normal tissues from 60 patients aged 18 years with TSCC diagnosed between January 2015 and December 2015 at the First People's Hospital of Yulin City, China. The Ethics Committee approved this study of the First People's Hospital of Yulin City (ID: YLSY‐IRB‐CR‐2020087). The experiments were undertaken with the understanding and written consent of each subject. Moreover, the study methodologies conformed to the standards set by the Declaration of Helsinki.

### Microarray data

Expression profile and clinical information data from four tongue cancer datasets (GSE13601, GSE31056 [10], GSE9844 [11], GSE30784 [12]) were downloaded from the GEO [13] database using the r software package geoquery [14] (v. 4.0.1); all samples were from *Homo sapiens*. GSE13601 is based on the GPL8300 ([HG_U95Av2] Affymetrix Human Genome U95 Version 2 Array) platform, GSE31056 is based on the GPL10526 ([HG‐U133_Plus_2] Affymetrix GeneChip Human Genome HG‐U133 Plus 2 Array [Brainarray Version 12]) platform, and GSE9844 and GSE30784 are based on the GPL570 ([HG‐U133_Plus_2] Affymetrix Human Genome U133 Plus 2.0 Array) platform. The GSE13601 dataset tissue was entirely tongue and included 30 tongue cancer and 25 normal samples. Only tongue tissue samples were retained from the GSE31056, including 11 tongue cancer and 12 normal samples. The GSE9844 dataset tissue was entirely tongue and included 26 tongue cancer and 12 normal samples. The GSE30784 dataset tissue was also entirely tongue and included 150 tongue cancer and 44 normal samples for analysis. We used the normal Between Arrays function in the limma [15] package to correct batch differences between the four datasets and obtained the gene expression matrices for each dataset. The corrected data were analyzed using the UMAP [16] package for dimensionality reduction. The TCGA public database of RNA expression was downloaded. The Genomics Data Commons TCGA Head and Neck Cancer cohort (v. 07‐19‐2019) [17] was incorporated in the analysis of samples, including 15 cases of tongue cancer and normal samples, and 129 cases without normal pairing.

### Identifying and functional analysis of differentially expressed genes (DEGs)

The limma package was used to screen the four datasets for DEGs, and we used the ggplot2 [18] package to generate volcano plots (adj. *P*‐value < 0.05 and |log2FC| > 1). We used the clusterProfiler [19] package to perform GO and KEGG enrichment analysis; adj. *P*‐value < 0.01 was considered statistically significant. The DESeq2 [20] package was used to screen the DEGs from the TCGA dataset and used the ggplot2 package to generate volcano plots to demonstrate DEG expression (adj. *P*‐value < 0.05 and |log2FC| > 2). We selected the 30 most up‐ and downregulated DEGs to display as a heatmap. GSEA [21] was performed on the gene expression matrix with the clusterProfiler package; we selected c2.cp.kegg.v7.0.symbols.gmt, c4.all.v7.2.symbols.gmt, c5.all.v7.2.symbols.gmt, and c8. All.V7.2. Symbols. Gmt as the reference gene sets. Each analysis was performed over 10,000 permutations. The normalized enrichment score (NES) and normal *P*‐values were calculated to classify the enrichment pathways of each phenotype. A false discovery rate < 0.25 was considered significantly enriched.

### Differential and ferroptosis‐related genes

The ferroptosis‐related gene list, totaling 259 genes, was downloaded from FerrDB [22]. The Venn diagrams were prepared with Venn Diagram to visualize the intersections of the significant gene sets within groups and then determined the intersection between DEGs and ferroptosis‐related genes.

### Receiver operating characteristic (ROC) analysis of ferroptosis‐related genes

Survival analysis data were only available from the GSE31056 dataset and 143 tongue cancer samples from TCGA. We analyzed the relationship between ferroptosis‐related genes and the overall survival (OS) of tongue cancer patients using the survival package [23]. ROC analyses were performed using the timeROC [24] package. The discriminative power of the model was assessed using the area under the ROC curve (ROC AUC).

### Construction of protein–protein interaction (PPI) network

cytoscape [25] software and the cytoHubba [26] plug‐in were used to construct and visualize the PPI; each node represents a gene or protein, and the edge between nodes represents the interaction of the molecules. We obtained 97 ferroptosis‐related genes using the string [27] (v. 10) database and construct PPI networks and analyze interactions between DEGs.

### Prognostic models were established using ferroptosis‐related genes

We used Cox univariate linear regression to analyze the relationship between ferroptosis‐related genes and the OS of tongue cancer patients in TCGA using the survival package. Survival curves were based on the Kaplan–Meier method. The hazard ratio (HR) was used to determine a gene variable's protective or harmful effects. A *P*‐value < 0.01 was considered statistically significant. Independent prognostic factors were identified using the Cox multivariate regression model. The prognostic model was then applied in the validation set GSE31056.

### Immunohistochemistry

For each patient, we carefully collected representative tissue cores of the TSCC tumor and adjacent section. Immunohistochemistry (IHC) staining was used to evaluate CA9 protein expression. Samples were fixed in 10% formalin for 24 h, embedded in paraffin, and cut in 5‐μm sections. Standard immunoperoxidase procedures were used to visualize CA9 in tumor and normal samples. Briefly, sections were deparaffinized, blocked with 3% H_2_O_2_, followed by incubation with anti‐CA9‐1 (Fuzhou Maixin Biotechnology Development Co. Ltd, Fuzhou, China) 60 min at 37 °C. After incubation with mice/rabbit (Fuzhou Maixin Biotechnology Development Co. Ltd), secondary antibody for 30 min, the sections were stained with DAB (Fuzhou Maixin Biotechnology Development Co. Ltd) for 5 min. Distilled water was used to stop color, hematoxylin counterstained after distilled water washed, dehydration, xylene transparent, neutral gum sealed.

### Statistical analysis

All statistical analyses were performed as the means + standard deviation and calculated in r (v. 3.4.1, Vienna, Austria). We used AUC analysis to investigate the predictive accuracy for each gene and Kaplan–Meier analysis evaluate the effect of a single factor on OS. HR was used to identify the protective or hazardous genes. A *P*‐value of less than 0.05 was considered statistically significant.

## Results

### Identification of DEGs in TSCC

**Fig. 1:**
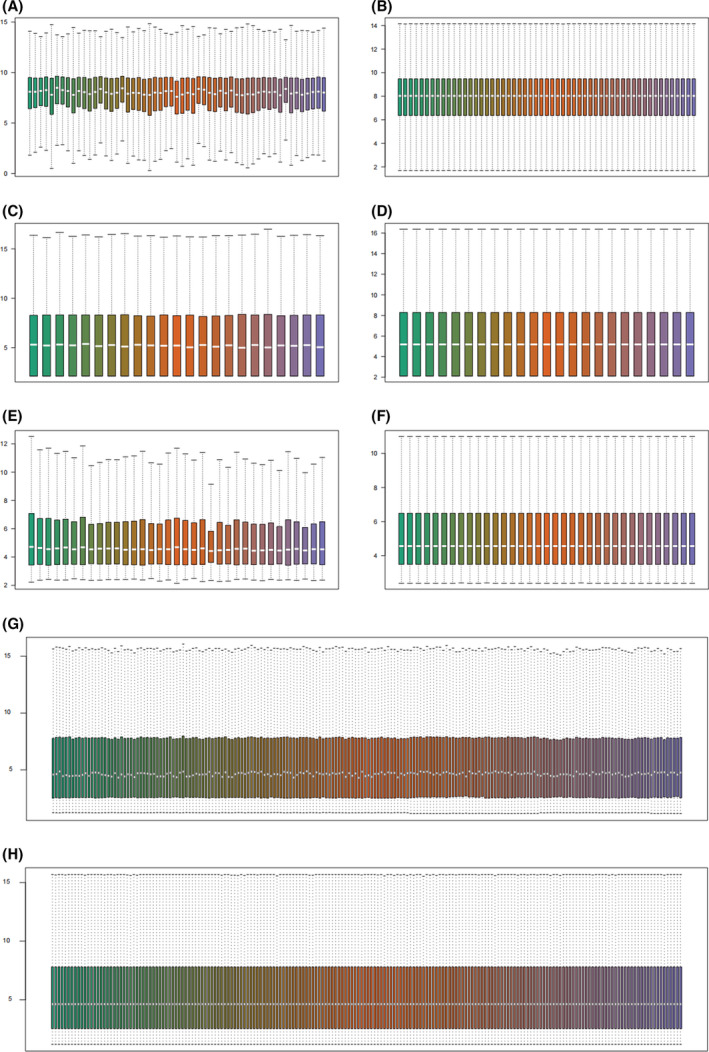
Four GEO databases with batch correction results (GSE13601, GSE31056, GSE9844, GSE30784). Expression distribution of GSE13601 (A) before and (B) after correction. Expression distribution of GSE31056 (C) before and (D) after correction. Expression distribution of GSE9844 (E) before and (F) after correction. Expression distribution of GSE30784 (G) before and (H) after correction.

**Fig. 2:**
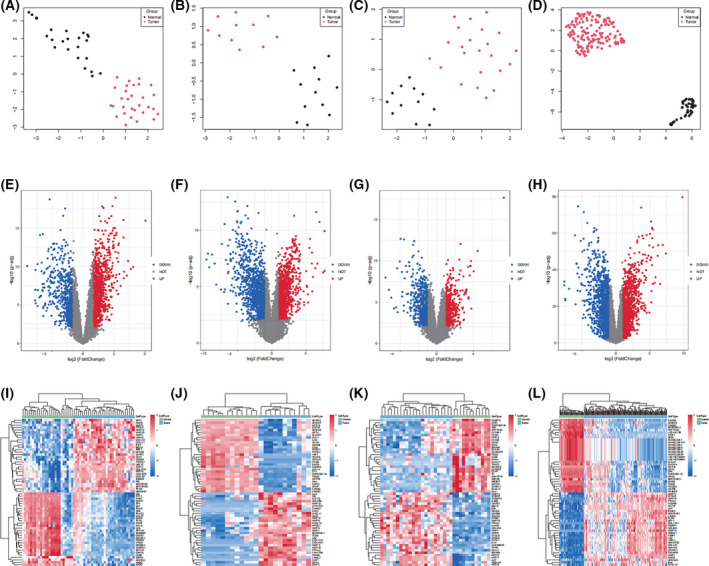
Results of difference analysis of four GEO datasets (GSE13601, GSE31056, GSE9844, GSE30784). PCA results of (A) GSE13601, (B) GSE31056, (C) GSE9844, and (D) GSE30784. Volcano plots for (E) GSE13601, (F) GSE31056, (G) GSE9844, and (H) GSE30784. Heatmaps for (I) GSE13601, (J) GSE31056, (K) GSE9844, and (L) GSE30784.

Four GEO datasets (GSE13601, GSE31056, GSE9844, GSE30784, Table 1) were normalized and batch effects within the group were removed (Fig. 1). After preprocessing, R was used to extract DEGs from the four gene expression matrices (Fig. 2A–D). As shown in the volcano plots, upregulated genes are depicted in red and downregulated genes in blue; then we selected the 30 most up‐ and downregulated DEGs and depicted them as a heatmap (Fig. 2E–L).

**Table 1:** Basic information about the GEO database (GSE13601, GSE31056, GSE9844, GSE30784).

Datasets Accession ID	Tumor	Control	Platforms ID	Platform length	Tissue
GSE13601	30	25	GPL8300	12625	Tongue
GSE31056	11	12	GPL10526	17788	Tongue
GSE30784	150	44	GPL570	54675	Tongue
GSE9844	26	12	GPL570	54675	Tongue

### Functional correlation analysis

**Fig. 3:**
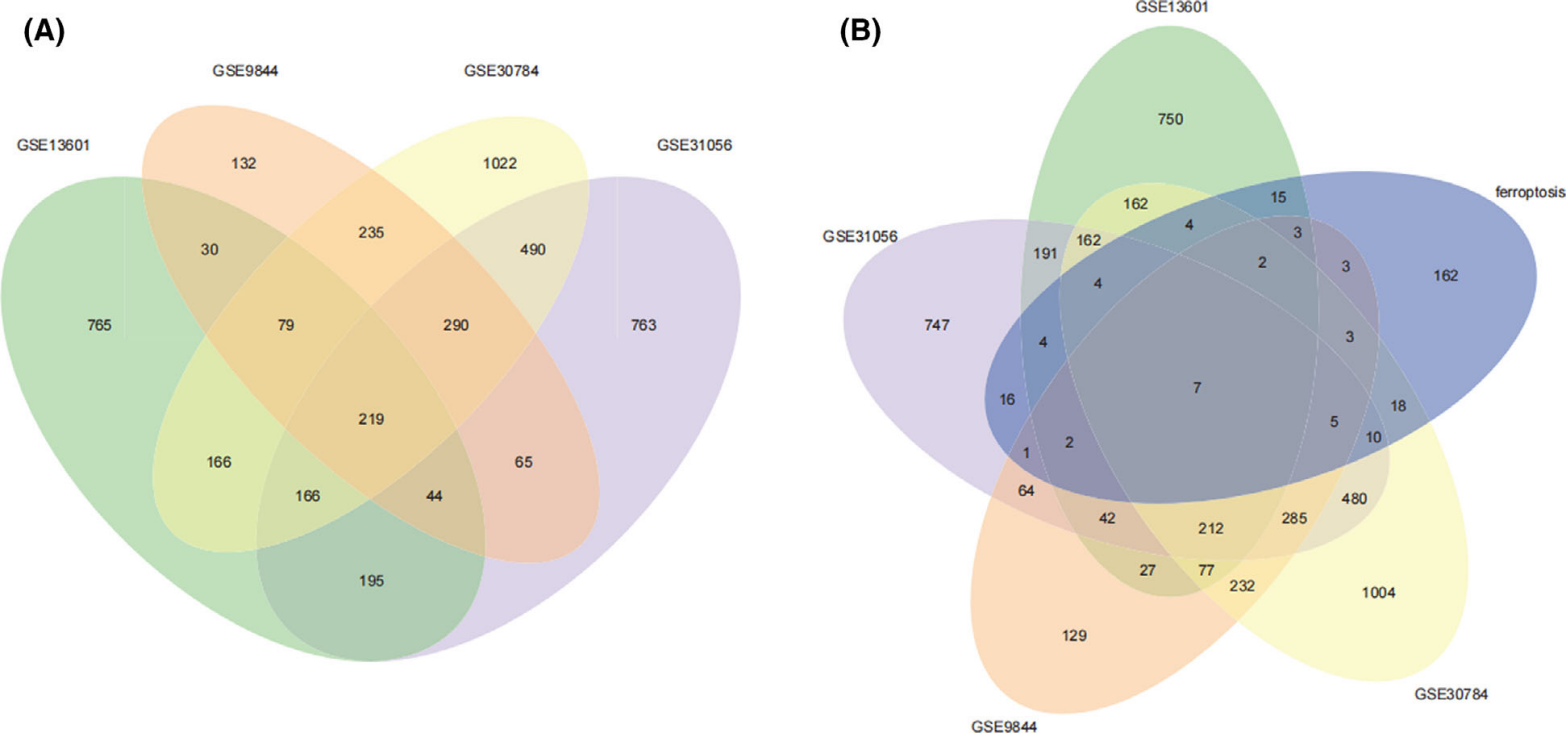
Differentially expressed and ferroptosis‐related genes in four GEO databases (GSE13601, GSE31056, GSE9844, GSE30784). Intersection of (A) differentially expressed and (B) ferroptosis‐related genes identified from the four GEO databases.

**Fig. 4:**
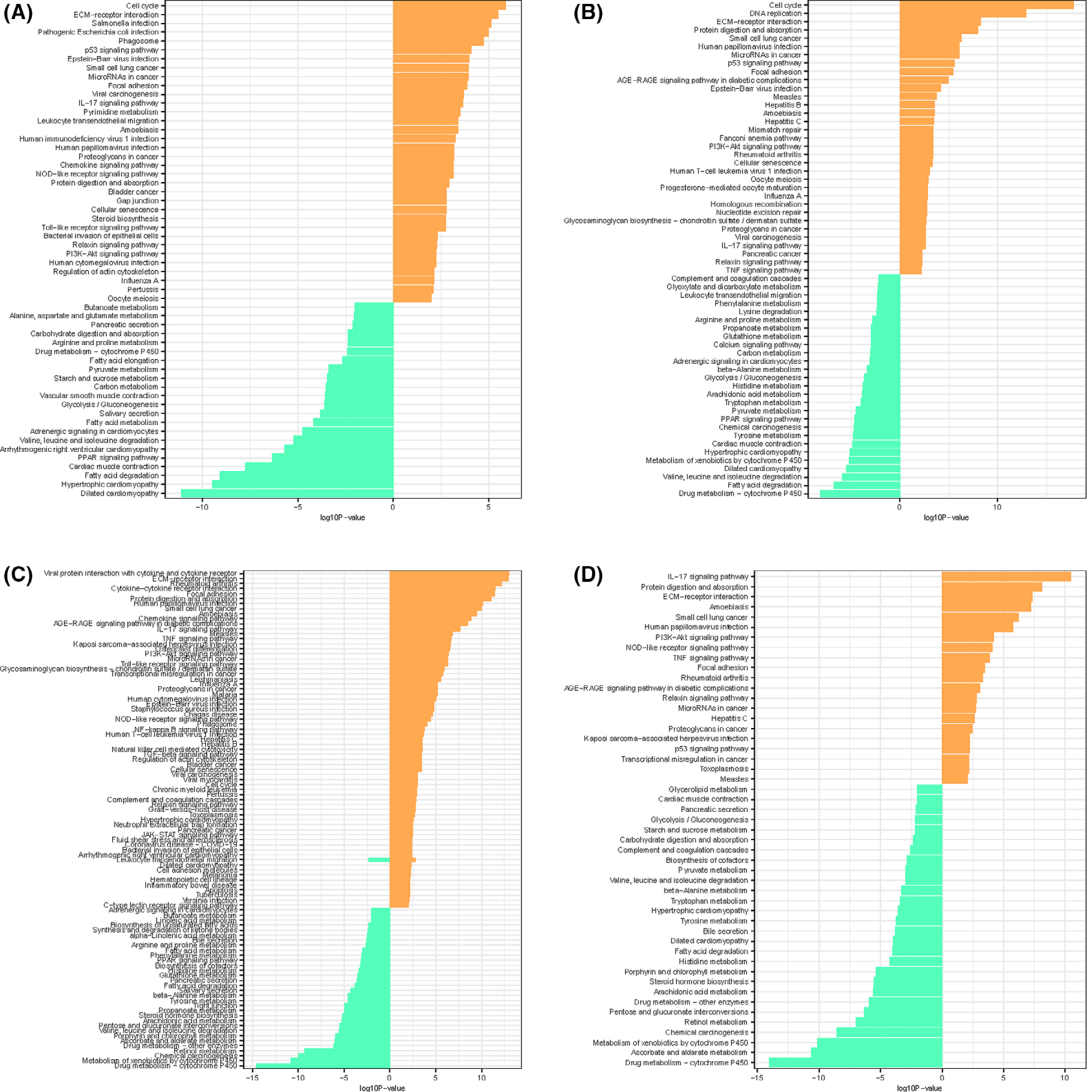
Results of KEGG enrichment analysis of differential expressed genes in (A) GSE13601, (B) GSE31056, (C) GSE9844, and (D) GSE30784.

We identified 219 DEGs in four GEO databases; 97 were ferroptosis‐related genes in the four datasets, and seven genes (*TNFAIP3*, *TP63*, *TFRC*, *LPIN1*, *NCF2*, *ALOX12*, and *TF*) overlapped in the five datasets (Fig. 3, Table S1). Based on these DEGs, KEGG pathway enrichment analysis results (Fig. 4, Table 2) showed that the extracellular matrix (ECM)‐receptor interaction, interleukin (IL)‐17, cell cycle, DNA replication, viral protein interaction with cytokine and cytokine receptor, and *p53* signaling pathways were significantly upregulated at the genetic level. GO term enrichment analysis of these DEGs, including cell component, molecular function, and biological process, were also analyzed (Table 3).

**Table 2:** KEGG pathway enrichment analysis of DEGs. GeneRatio: The numerator is the number of genes enriched to this GO entry, and the denominator is genes from differential expression analysis. BgRatio: Background Ratio, the denominator is the number of genes that have GO annotations in all the protein‐coding genes in human, the numerator is the number of genes in these genes that are commented onto this GO entry. Q value: *P*‐value after correction. Count: the number of genes enriched to this GO entry from the input gene for enrichment analysis. *Noun explanation.

ID	GeneRatio^*^	BgRatio^*^	Q value^*^	Count^*^	Group
hsa00982	20/248	63/7179	2.39475E‐12	20	−1
hsa00053	12/248	27/7179	2.97253E‐09	12	−1
hsa00980	17/248	68/7179	6.23582E‐09	17	−1
hsa04657	19/219	94/7179	8.12551E‐09	19	1
hsa05204	16/248	74/7179	1.73837E‐07	16	−1
hsa04974	17/219	101/7179	8.86914E‐07	17	1
hsa05146	16/219	102/7179	3.50938E‐06	16	1
hsa04512	15/219	88/7179	3.50938E‐06	15	1
hsa00830	13/248	61/7179	5.33227E‐06	13	−1
hsa00040	9/248	30/7179	1.86991E‐05	9	−1
hsa05222	14/219	92/7179	2.84334E‐05	14	1

**Table 3:** GO enrichment analysis of DEGs. GeneRatio: The numerator is the number of genes enriched to this GO entry, and the denominator is genes from differential expression analysis. BgRatio: Background Ratio, the denominator is the number of genes that have GO annotations in all the protein‐coding genes in humans, the numerator is the number of genes in these genes that are commented onto this GO entry. Q value: *P*‐value after correction. Count: the number of genes enriched to this GO entry from the input gene for enrichment analysis. *Noun explanation.

ID	GeneRatio^*^	BgRatio^*^	Q value^*^	Count^*^
GO:0005201	81/2209	165/16019	1.02096E‐24	81
GO:0005201	43/915	165/16019	1.24093E‐14	43
GO:0003779	119/2209	421/16019	1.30146E‐12	119
GO:0003779	113/1905	397/13947	3.39983E‐12	113
GO:0005201	60/1905	160/13947	1.37671E‐11	60
GO:0003779	98/1475	273/8558	3.36782E‐11	98
GO:0003779	65/915	421/16019	6.97361E‐11	65
GO:0008307	26/1905	42/13947	1.61449E‐10	26
GO:0008009	28/2209	48/16019	2.20772E‐10	28
GO:0005518	33/2209	66/16019	5.24354E‐10	33
GO:0042379	32/2209	63/16019	5.24354E‐10	32

### Prognostic significance of ferroptosis‐related genes

**Fig. 5:**
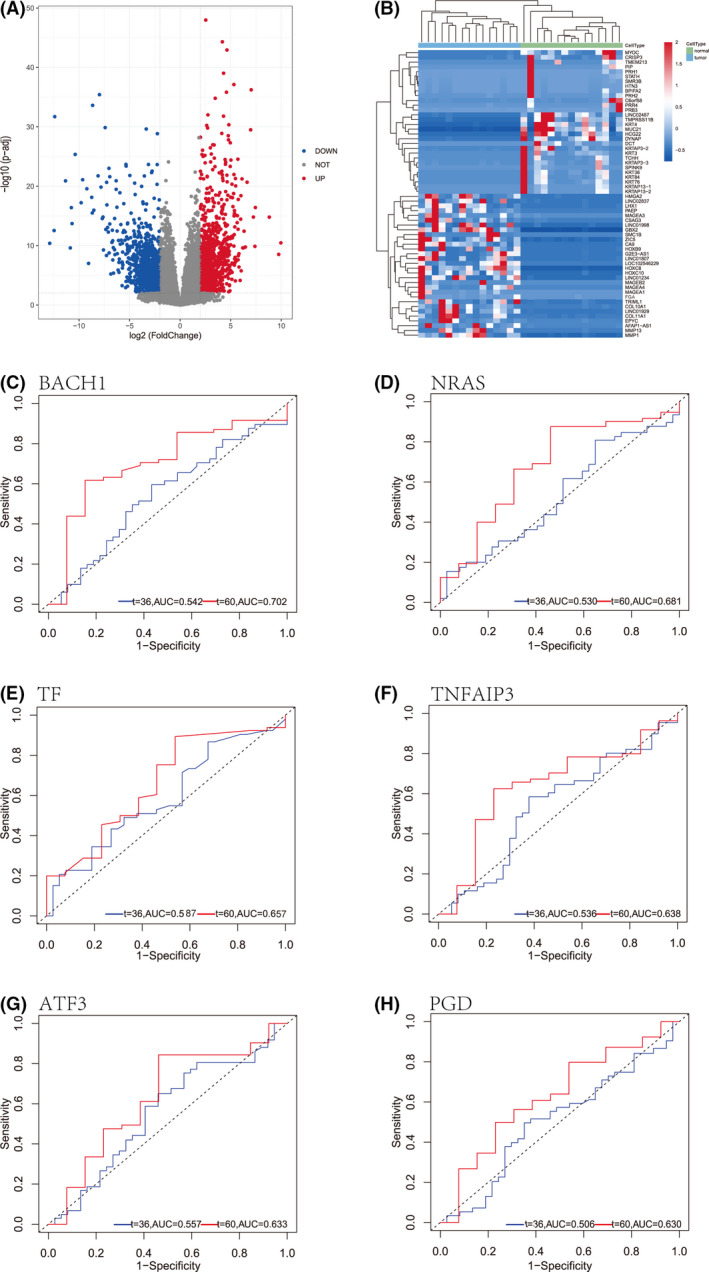
Results of differentially expressed genes and ROC analysis in TCGA. (A) Volcano plot. (B) Heatmap. ROC analysis of (C) BACH1, (D) NRAS, (E) TF, (F) TNFAIP3, (G) ATF3, and (H) PGD.

**Fig. 6:**
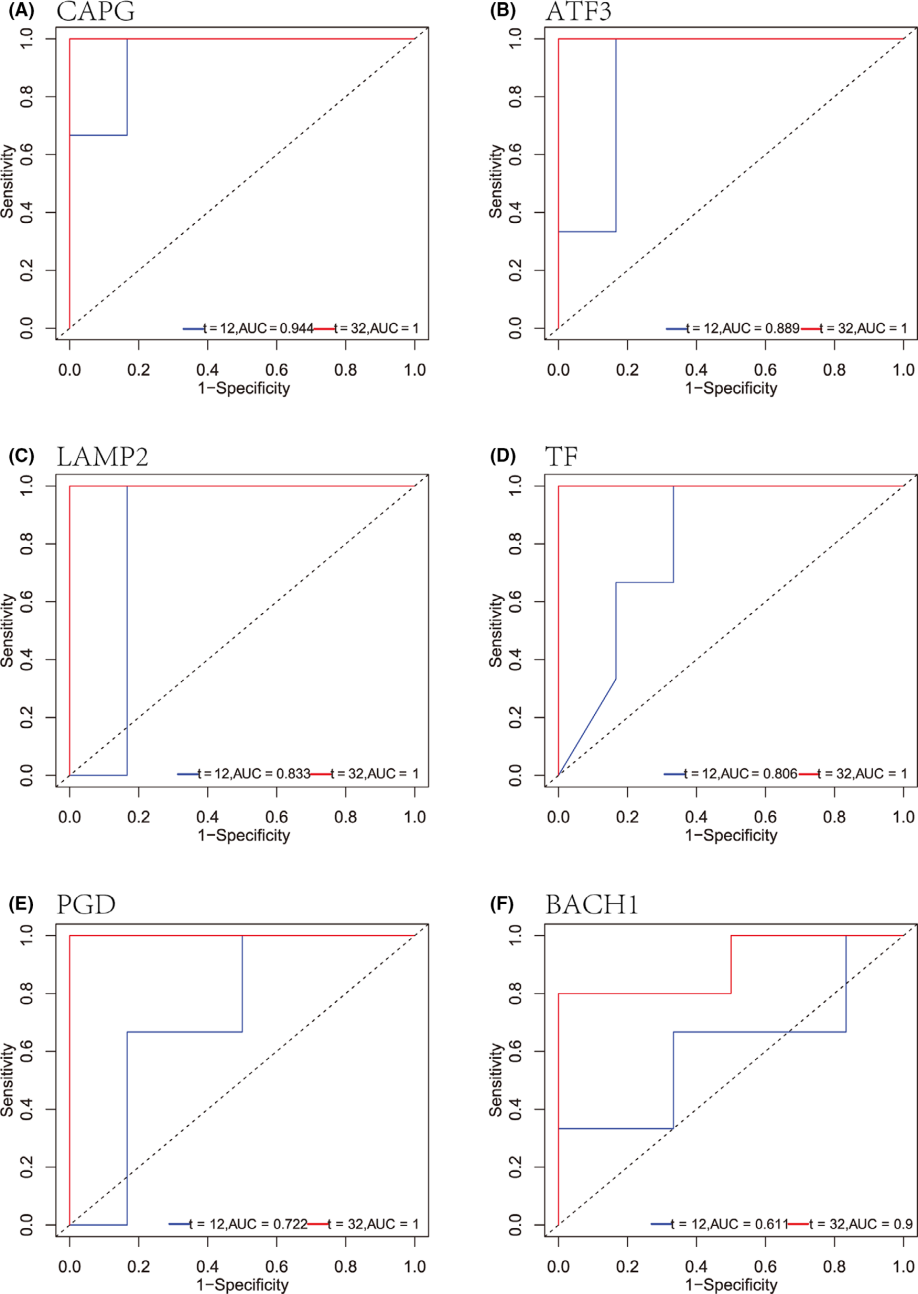
ROC analysis of ferroptosis‐related differentially expressed genes in GSE31056. (A) CAPG, (B) ATF3, (C) LAMP2, (D) TF, (E) PGD, and (F) BACH1.

DEGs were extracted from the gene expression matrix of TCGA and displayed as a volcano plot, with upregulated genes depicted in red and downregulated genes in blue (Fig. 5A). The 30 most up‐ and downregulated DEGs are displayed as a heatmap (Fig. 5B) to show the pathway enrichment. ROC analysis of TCGA data showed that the BACH1, NRAS, TF, TNFAIP3, ATF3, and PGD genes were associated with survival in TSCC (Fig. 5C–H). ROC analysis of the GSE31056 dataset showed that BACH1, TF, ATF3, PGD, CAPG, and LAMP2 were associated with survival (Fig. 6A–F).

### GSEA and GSVA in TCGA

**Fig. 7:**
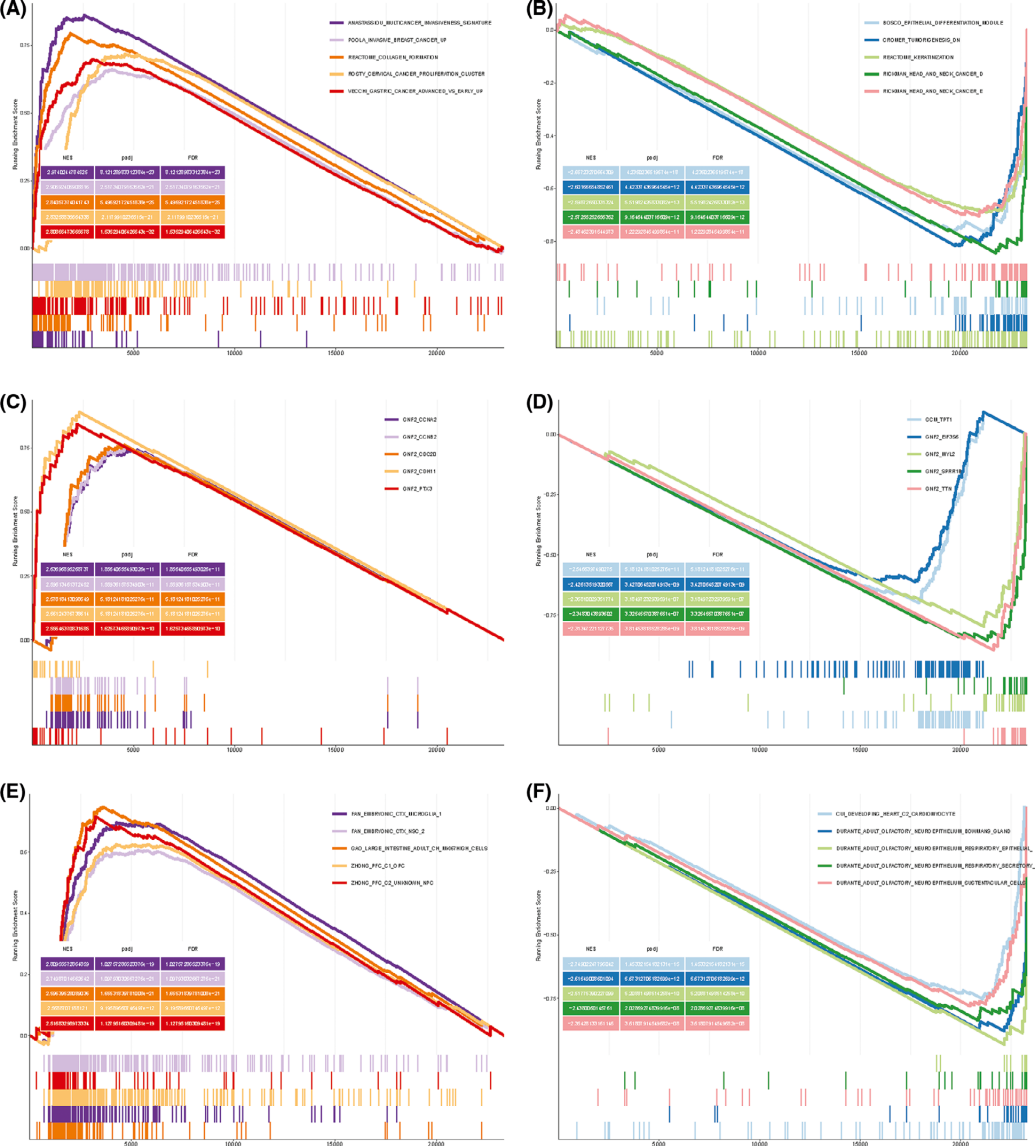
GSEA results from TCGA data. The five most upregulated pathways in (A) C2, (C) C4 and, (E) C8. The five most downregulated pathways in (B) C2, (D) C4, and (F) C8.

**Fig. 8:**
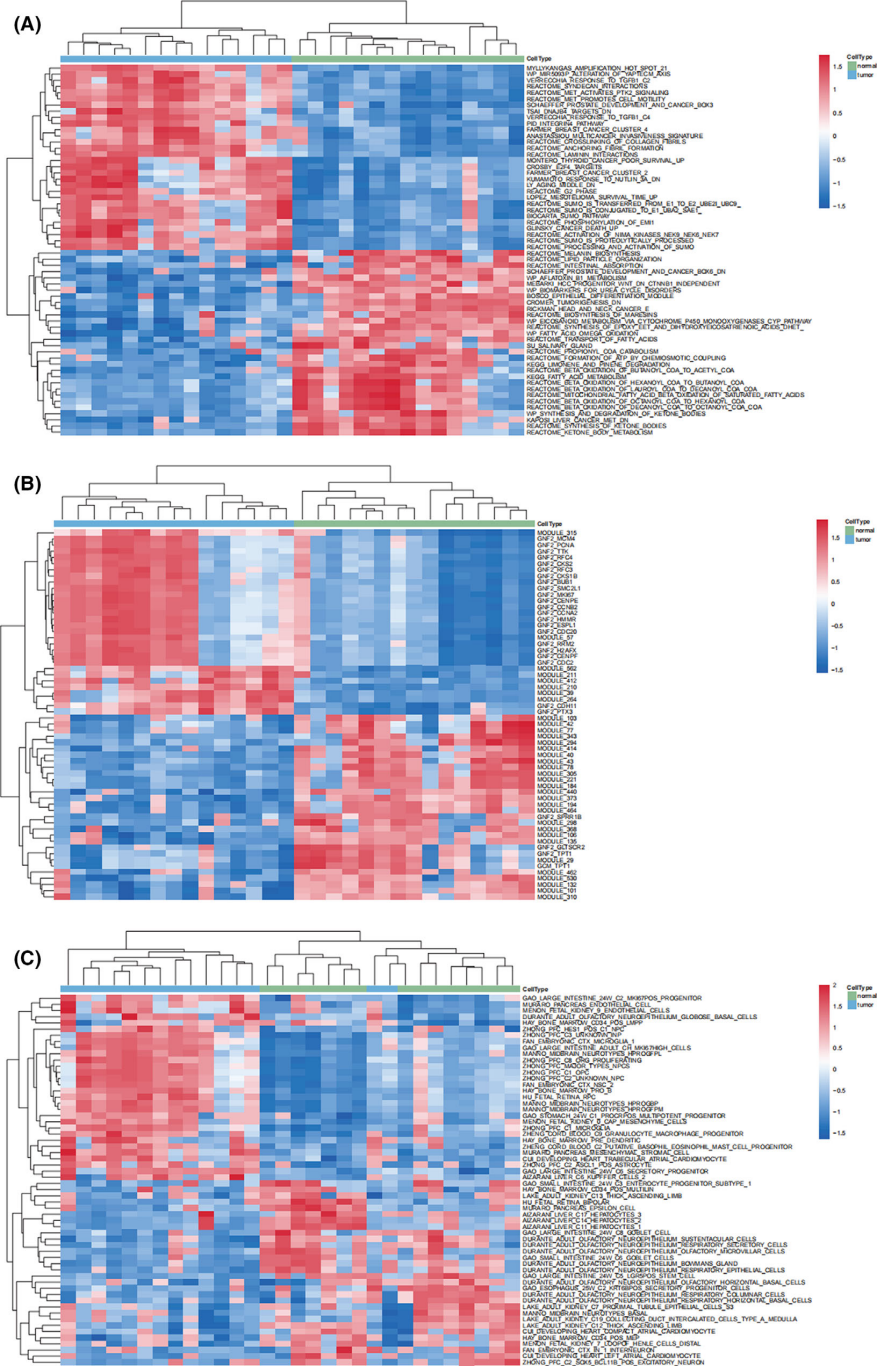
GSVA results from TCGA data. Heatmaps of pathway differences in (A) C2, (B) C4, and (C) C8.

We next performed GSEA on TCGA results to show the most significant enrichment signaling pathway (Fig. 7, Table 4) based on the NES for GSEA in C2 (curated gene sets), C4 (computational gene sets), and C8 (cell type signature gene sets). We then conducted gene set variation analysis (GSVA) to identify pathway differences in C2, C4, and C8 (Fig. 8). We observed that KAPOSI_LIVER_CANCER_MET_DN was significantly downregulated in C2 (logFC < −1, *P*‐value < 0.001), whereas the MYLLYKANGAS_AMPLIFICATION_HOT_SPOT_21 pathway was upregulated (logFC > 1, *P*‐value < 0.001).

**Table 4:** GSEA. setSize: The GO entry contains the number of genes in the expression dataset. NES, normalized ES value after correction. *Noun explanation.

Description	setSize^*^	Enrichmentscore	NES^*^	q values
GO_CONTRACTILE_FIBER	233	−0.736572236	−2.864296268	3.88227E‐34
SHEDDEN_LUNG_CANCER_POOR_SURVIVAL_A6	456	0.587252775	2.547111569	4.95042E‐31
SENGUPTA_NASOPHARYNGEAL_CARCINOMA_DN	347	−0.638501234	−2.58275205	2.47509E‐27
KOBAYASHI_EGFR_SIGNALING_24HR_DN	251	0.6449726	2.665943347	1.06526E‐24
GO_MUSCLE_SYSTEM_PROCESS	437	−0.579691185	−2.392701988	6.65876E‐24
VECCHI_GASTRIC_CANCER_EARLY_DN	375	−0.604772494	−2.453764439	2.50443E‐23
SENGUPTA_NASOPHARYNGEAL_CARCINOMA_UP	301	0.609228653	2.549171527	2.84686E‐23
ZHONG_PFC_C1_OPC	236	0.643225619	2.638172811	3.70056E‐23
GO_I_BAND	138	−0.760987059	−2.801318495	1.067E‐22
FAN_EMBRYONIC_CTX_MICROGLIA_1	154	0.704237279	2.719531242	1.03723E‐21
ROSTY_CERVICAL_CANCER_PROLIFERATION_CLUSTER	140	0.733037178	2.826858911	1.43168E‐21

### PPI network construction

**Fig. 9:**
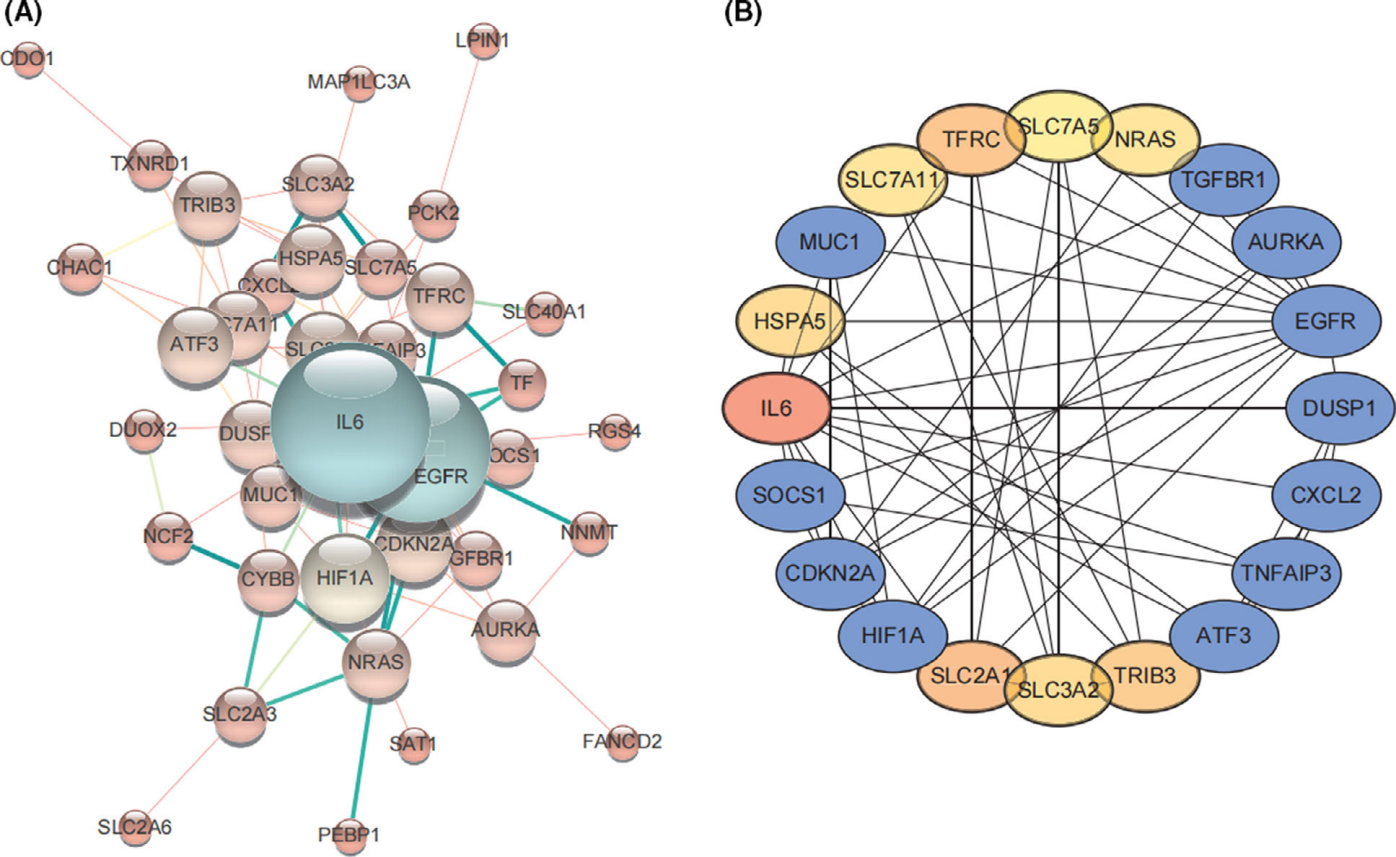
Protein–protein interaction (PPI) network analysis. (A) PPI network of ferroptosis‐related genes generated using the STRING database. (B) Analysis of the top 20 hub genes with maximum correlation criteria using cytoHubba.

A total of 97 ferroptosis‐related genes were plotted on the STRING (v. 10) website and displayed using Cytoscape (Fig. 9A). The cytoHubba [26]plug‐in was used to calculate and visualize the top 20 genes (Fig. 9B). These results showed four genes in the SLC family. In addition, NRAS and TNFAIP3 of the top 20 genes were overlapped by univariate COX regression analysis of OS prognosis in the TCGA database.

### Construction and verification of prediction model

**Fig. 10:**
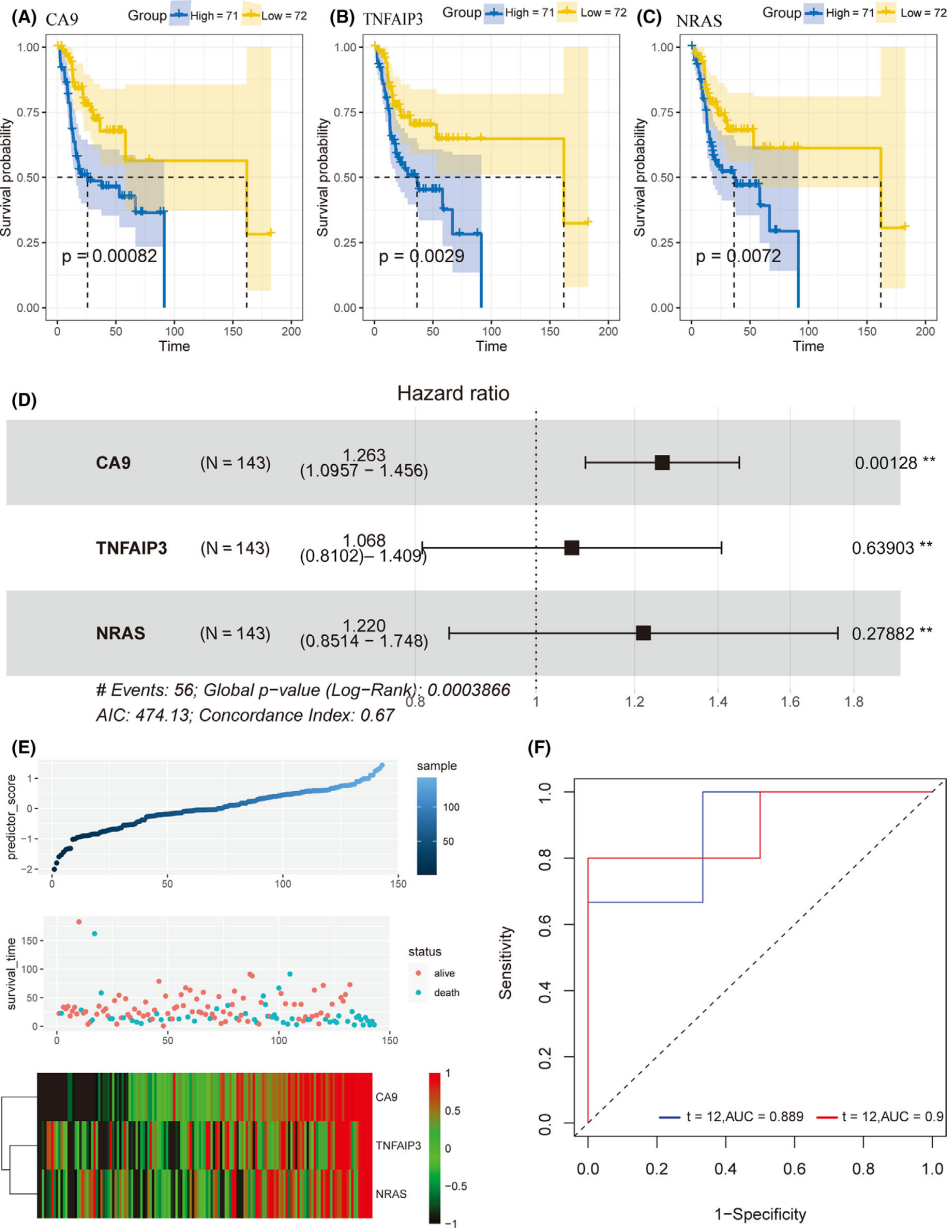
Multivariate Cox regression prognostic model established from the TCGA database. Three genes, (A) CA9, (B) TNFAIP3, and (C) NRAS, were significantly related to overall survival (OS) in a Cox regression model of TCGA data. (D) Forest plot representing multivariate analysis of OS related to the genes from (A–C). (E) From top to bottom, risk prediction model score, sample survival, and the expression levels of CA9, TNFAIP3, and NRAS in the samples. (F) ROC plot from the validation dataset GSE31056 showing the predictive efficacy of the multivariate Cox disease‐specific survival model.

We first performed univariate Cox regression analysis of OS prognosis using the survival package in R in matched tumor and normal control samples from TCGA (*P* < 0.01). We selected three genes (*P* ≤ 0.001) associated with survival (Fig. 10A–D, Table 5), CA9, TNFAIP3, and NRAS (Table S2); then we constructed a prognostic model based on their expression. We further developed this prognostic model using multivariate Cox regression analysis (Fig. 10E) Equation (1):

(1) Riskscore=‐0.95(CA9)‐0.83(TNFAIP3)‐0.75(NRSA)

**Table 5:** COX related information.

Gene	coef	HR (95% CI for HR)	*P* value
TNFAIP3	−0.83	0.43 (0.25–0.76)	0.0038
NRAS	−0.75	0.47 (0.27–0.83)	0.0087
CA9	−0.95	0.39 (0.22–0.69)	0.0012

The calibration graph in the validation set GSE31056 was used to test the prediction efficiency of the model (Fig. 10F). The results indicated that the OS prognostic model constructed could better predict 3‐year OS.

### The expression of CA9 was determined by IHC

**Fig. 11:**
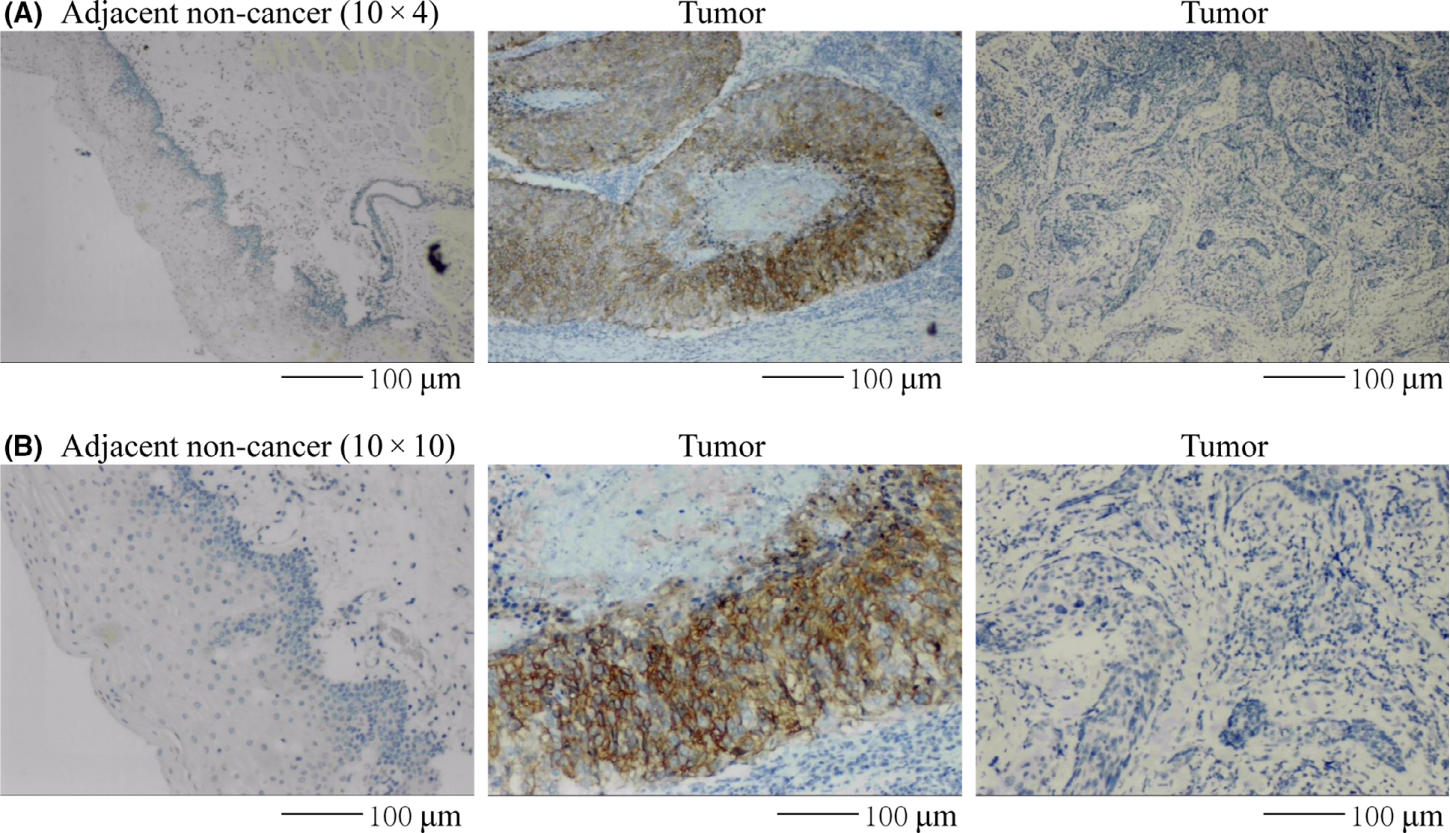
CA9 immunohistochemical staining in selected cases of TSCC. (A,B) Low and high CA9 staining in adjacent tissues and tongue cancer tissues.

The previous bioinformatics found CA9 high expression in TSCC, and our findings on CA9 and TSCC survival prompted us to validate CA9 expression in TSCC further. We examined the specimens from 60 independent patients and compared CA9 expression between the tongue cancer and adjacent tissues by immunohistochemistry (IHC) analysis. Consistent with previous reports, CA9 staining was negative in the cytoplasmic of adjacent tissue cells; meanwhile, CA9 staining was strongly positive in the cancer tissue cells (Fig. 11A,B, Table 6). IHC analysis revealed that the expression level of CA9 in the tongue cancer tissues was significantly enhanced.

**Table 6:** Clinical baseline data for CA9 immunohistochemical assay.

Characteristic	Age		Gender		Stage		Pathologic T‐stage	
Subtype	< 60	≥ 61	Male	Female	Stage I–II	Stage III–IV	T1 + T2	T3 + T4
No. of cases	25	35	32	28	27	33	41	19

## Discussion

TSCC is the most common malignancy in the oral cavity; despite advances in diagnosis and treatment, the prognosis of advanced states has not significantly improved [28]. The reported 5‐year rates of disease‐free survival ranged from 30% to 72% for the younger cohorts (≤45 years ) and 42% to 81% for the older cohorts [29], and the etiology of tongue cancer, especially the molecular mechanism, remains unclear. Ferroptosis may play a key role in tumor suppression and is a potential target for cancer therapy. An evaluation of ferroptotic cellular mechanisms and pathophysiological environments suggests that its regulation may be beneficial in treating cancer [30]. Although many experiments have shown that promoting ferroptosis is beneficial to cancer treatment, the research on ferroptosis in tongue cancer is insufficient, and the molecular mechanism of ferroptosis in tongue cancer needs to be explored further. In this study, we analyzed biological processes involving ferroptosis‐related genes using GO/KEGG/GSEA and evaluated the diagnostic value of using ferroptosis‐related genes for TSCC underlying GEO, FerrDB, and TCGA,. Then we performed IHC detection of CA9, where multivariate analysis is significant, and confirmed that it was highly expressed in tumor tissues. A prognostic model developed based on ferroptosis‐related genes showed a good predictive ability for OS of TSCC. Our results suggested that ferroptosis was closely related to TSCC and may affect TSCC prognosis through effects on the ECM‐receptor interaction and IL‐17 signaling pathways.

We analyzed the DEGs by KEGG/GO. The results showed that genes in the ECM‐receptor interaction and IL‐17 signaling pathways were significantly upregulated. Specific interactions between cells and ECM are mediated by transmembrane molecules that directly or indirectly control cell activities, such as adhesion, migration, differentiation, proliferation, and apoptosis. A study on gastric cancer and ferroptosis showed that the mortality risk score was associated with the ECM‐receptor interaction pathway and tumor immunity [31]. In addition, the transcriptome analysis of 72 oral squamous cell carcinoma of the gingivo‐buccal region (OSCC‐GB) patients from multiple hospitals showed that the ECM‐receptor interaction pathway was significantly enriched in tumor tissues [32]. With the deepening of research, it has been found that proinflammatory cytokine IL‐17 is closely related to breast cancer, which plays an essential role in promoting tumor proliferation, invasion, and metastasis and is significantly associated with poor prognosis [33]. Studies on liver cancer have confirmed that erastin, an inducer of ferroptosis, inhibits liver cancer cell proliferation and progression, and bioinformatics analysis showed that erastin affected differentiation of Th17 cells and the IL‐17 signaling pathway [34]. Taken together, our results, along with previous studies, suggest that ferroptosis may be associated with the progression of tongue cancer through the ECM‐receptor interaction and IL‐17 signaling pathways.

Next, we explored the prognostic value of ferroptosis‐related genes in tongue cancer. We selected eight ferroptosis‐related genes (BACH1, NRAS, TF, TNFAIP3, ATF3, PGD, CAPG, and LAMP2) to predict survival and used AUC to determine their predictive ability. These genes were associated with the ECM‐receptor interaction and IL‐17 signaling pathways [35, 36, 37, 38, 39]. The genes mentioned above showed good predictive ability at the 5‐year and 32‐month survival. As shown in Table 5, univariate Cox regression analysis showed that CA9 (HR = 0.39 [0.22–0.69], *P* = 0.0012), TNFAIP3 (HR = 0.43 [0.25‐–0.76], *P* = 0.0038), and NRAS (HR = 0.47 [0.27–0.83], *P* = 0.0087) were significantly related to TSCC prognosis, with higher expression of these genes indicating poorer prognosis. After we included the three genes in multivariate Cox analysis, CA9 was still an independent predictor (HR = 1.263 [1.0957–1.456], *P* = 0.00128). In addition, through IHC, we found that CA9 was strongly positive in tongue cancer tissues but negative in adjacent tissues. Therefore, we used these genes to construct a prognosis model. Other studies have verified the predictive value of the genes used in our prediction model. CA9 can not only be used as a prognostic marker for bladder cancer [40], previous studies have shown that high CA9 expression is associated with poor prognosis of TSCC [41]. Moreover, CA9 confers resistance to ferroptosis in malignant mesothelioma under hypoxia [42]. HRAS, NRAS, and KRAS, collectively referred to as oncogenic RAS, are the most frequently mutated driver proto‐oncogenes in cancer; the relationship between oncogenic RAS and ferroptosis is still controversial [43]. As the phosphorylation gene of MAPK protein, NRAS has become one of the taxonomic markers of four subtypes of melanoma [44]; moreover, high expression of NRAS is associated with poor prognosis of lung adenocarcinoma [45]. Tumor extracellular vesicles carrying miR‐125b‐5p enter diffuse large B‐cell lymphoma (DLBCL) cells and target TNFAIP3, thereby reducing the sensitivity of DLBCL to rituximab [46], and overexpression of TNFAIP3 is associated with a lower survival rate in breast cancer patients [47]; a study about ferroptosis after cerebral hemorrhage also find TNFAIP3 upregulation [48]. Our model predicted TSCC patient survival in the validation cohorts at 1 year (AUC = 0.889) and 32 months (AUC = 0.9). Therefore, our prognostic model may become a valuable predictive method in the future, and we intended to include detailed clinical records for further refinement.

This study combined the identification of DEGs with ferroptosis‐related genes and generated a PPI network in the STRING database with the 97 ferroptosis‐related genes utilized in this study. The top 20 genes belonged to the SLC family, consistent with previous studies [9]. The SLC family is associated with the proliferation and migration of tumors, IL‐17 signaling [49], and has also been shown to drive tumor metastasis [50]. Other genes among the top 20, including ATF3, TGFBR1, and EGFR, are associated with the ECM‐receptor interaction and IL‐17 signaling pathways [51]. The PPI analysis suggests that ferroptosis‐related genes are associated with the progression of TSCC and further confirmed that ferroptosis‐related genes might influence prognosis through ECM‐receptor interaction and IL‐17 signaling.

Inevitably, this study has some limitations. First, this was a retrospective study because the data sources were TCGA and GEO. Second, the exact mechanism by which the three genes in our model influence TSCC are unknown. Third, large, clinical, multicenter studies are needed to validate our model. Despite the above shortcomings, the results suggest that our model can be used as a reliable prognostic predictor of TSCC survival.

## Conclusion

In conclusion, ferroptosis was closely related to TSCC and ferroptosis‐related‐genes may affect the prognosis of TSCC through the ECM‐receptor interaction and the IL‐17 signaling pathways. Additionally, we screened genes as potential prognostic markers and constructed a prognostic model based on these ferroptosis‐related genes. However, more experiments are needed to validate the current findings further. Our study may provide a broader idea for exploring the molecular mechanism and therapeutic targets of TSCC.

## Conflict of interest

The authors declare no conflicts of interest.

## Author contributions

JHZ and LFL: conceptualization. HSZ and YZT: investigation. HSZ and YZT: data curation. HSZ and YZT: writing—original draft preparation. QWH and ZMC: methodology: LJJ and HLY: software. JHZ and LFL: funding acquisition. All authors contributed to article revision, read, and approved the submitted version.

## Supporting information


**Table S1**. 259 Ferroptosis‐related genes from the FerrDB database.

**Table S2**. Expression information of CA9, TNFAIP3, and NRAS.

## Data Availability

The datasets for this study can be found in the GEO (https://www.ncbi.nlm.nih.gov/geo/), TCGA (https://gdc.xenahubs.net/), and FerrDB (http://www.zhounan.org/ferrdb/) databases. The analyzed data for this study can be found in the R software (v. 4.0.1, http://r‐project.org/) and STRING (http://string‐db.org, version 10).
