# Supplementary material for: Identification of ferroptosis‐related genes as potential biomarkers of tongue squamous cell carcinoma using an integrated bioinformatics approach
Source: FEBS Open Bio. 2021 Dec 24;12(2):412–29. doi: 10.1002/2211-5463.13348 (PMC8804613; doi:10.1002/2211-5463.13348)
Supplement: Supplementary file 1 — Table S1. 259 Ferroptosis‐related genes from the FerrDB database. [file FEB4-12-412-s001.docx]

Supplementary Table 1 259 Ferroptosis-related genes from the FerrDB database

| Symbol | Name | HGNC_ID | PMID |
| --- | --- | --- | --- |
| ACSF2 | Acyl-CoA synthetase family member 2 | HGNC:26101 | 22632970 |
| ATP5MC3 | ATP synthase membrane subunit c locus 3 | HGNC:843 | 22632970 |
| CS | Citrate synthase | HGNC:2422 | 22632970 |
| CYBB | Cytochrome b-245 beta chain | HGNC:2578 | 22632970 |
| DUOX1 | Dual oxidase 1 | HGNC:3062 | 22632970 |
| DUOX2 | Dual oxidase 2 | HGNC:13273 | 22632970 |
| EMC2 | ER membrane protein complex subunit 2 | HGNC:28963 | 22632970 |
| G6PD | Glucose-6-phosphate dehydrogenase | HGNC:4057 | 22632970 |
| IREB2 | Iron response element binding protein 2 | HGNC:6115 | 22632970 |
| NOX1 | Nicotinamide adenine dinucleotide phosphate (NADPH) oxidase (NOX) 1 | HGNC:7889 | 22632970 |
| NOX3 | Nicotinamide adenine dinucleotide phosphate (NADPH) oxidase (NOX) 3 | HGNC:7890 | 22632970 |
| NOX4 | Nicotinamide adenine dinucleotide phosphate (NADPH) oxidase (NOX) 4 | HGNC:7891 | 22632970 |
| NOX5 | Nicotinamide adenine dinucleotide phosphate (NADPH) oxidase (NOX) 5 | HGNC:14874 | 22632970 |
| PGD | Phosphoglycerate dehydrogenase | HGNC:8891 | 22632970 |
| RPL8 | Ribosomal protein L8 | HGNC:10368 | 22632970 |
| SLC7A11 | Solute carrier family 7 member 11 | HGNC:11059 | 22632970 |
| VDAC2 | Valtage-dependent anion channels 2 | HGNC:12672 | 22632970 |
| GPX4 | Glutathione peroxidase 4 | HGNC:4556 | 24439385 |
| FLT3 | Fms related tyrosine kinase 3 | HGNC:3765 | 24739485 |
| PIK3CA | Phosphatidylinositol-4,5-bisphosphate 3-kinase catalytic subunit alpha | HGNC:8975 | 24739485 |
| AKR1C1 | Aldo-keto reductase family 1 member C1 | HGNC:384 | 24844246 |
| AKR1C2 | Aldo-keto reductase family 1 member C2 | HGNC:385 | 24844246 |
| AKR1C3 | Aldo-keto reductase family 1 member C3 | HGNC:386 | 24844246 |
| SCP2 | Sterol carrier protein 2 | HGNC:10606 | 25402683 |
| RB1 | RB transcriptional corepressor 1 | HGNC:9884 | 25444922 |
| HSF1 | Heat shock transcription factor 1 | HGNC:5224 | 25728673 |
| HSPB1 | Heat shock protein family B (small) member 1 | HGNC:5246 | 25728673 |
| TP53 | Tumor protein p53 | HGNC:11998 | 25799988 |
| ACSL4 | Acyl-CoA synthetase long chain family member 4 | HGNC:3571 | 25965523 |
| LPCAT3 | Lysophosphatidylcholine acyltransferase 3 | HGNC:30244 | 25965523 |
| HRAS | HRas proto-oncogene, GTPase | HGNC:5173 | 26157704 |
| KRAS | KRAS proto-oncogene, GTPase | HGNC:6407 | 26157704 |
| NRAS | NRAS proto-oncogene, GTPase | HGNC:7989 | 26157704 |
| GCLC | Glutamate-cysteine ligase catalytic subunit | HGNC:4311 | 26166707 |
| GLS2 | Glutaminase 2 | HGNC:29570 | 26166707 |
| GOT1 | Glutamic-oxaloacetic transaminase 1 | HGNC:4432 | 26166707 |
| SLC1A5 | Solute carrier family 1 member 5 | HGNC:10943 | 26166707 |
| SLC38A1 | Solute carrier family 38 member 1 | HGNC:13447 | 26166707 |
| TF | Transferrin | HGNC:11740 | 26166707 |
| TFR2 | Transferrin receptor 2 | HGNC:11762 | 26166707 |
| TFRC | Transferrin receptor | HGNC:11763 | 26166707 |
| CARS1 | Cysteinyl-tRNA synthetase 1 | HGNC:1493 | 26184909 |
| ALOX5 | Arachidonate 5-lipoxygenase | HGNC:435 | 26235588 |
| FTH1 | Ferritin heavy chain 1 | HGNC:3976 | 26403645 |
| KEAP1 | Kelch like ECH associated protein 1 | HGNC:23177 | 26403645 |
| NFE2L2 | Nuclear factor, erythroid 2 like 2 | HGNC:7782 | 26403645 |
| NQO1 | NAD(P)H quinone dehydrogenase 1 | HGNC:2874 | 26403645 |
| SQSTM1 | Sequestosome 1 | HGNC:11280 | 26403645 |
| HMOX1 | Heme oxygenase 1 | HGNC:5013 | 26405158 |
| MUC1 | Mucin 1, cell surface associated | HGNC:7508 | 26930718 |
| SLC3A2 | Solute carrier family 3 member 2 | HGNC:11026 | 26945935 |
| MT1G | Metallothionein 1G | HGNC:7399 | 27015352 |
| ATG5 | Autophagy related 5 | HGNC:589 | 27245739 |
| ATG7 | Autophagy related 7 | HGNC:16935 | 27245739 |
| NCOA4 | Nuclear receptor coactivator 4 | HGNC:7671 | 27245739 |
| SLC40A1 | Solute carrier family 40 member 1 | HGNC:10909 | 27441659 |
| ALOX12 | Arachidonate 12-lipoxygenase, 12S type | HGNC:429 | 27506793 |
| ALOX12B | Arachidonate 12-lipoxygenase, 12R type | HGNC:430 | 27506793 |
| ALOX15 | Arachidonate 15-lipoxygenase | HGNC:433 | 27506793 |
| ALOX15B | Arachidonate 15-lipoxygenase type B | HGNC:434 | 27506793 |
| ALOXE3 | Arachidonate lipoxygenase 3 | HGNC:13743 | 27506793 |
| PHKG2 | Phosphorylase kinase catalytic subunit gamma 2 | HGNC:8931 | 27506793 |
| CISD1 | CDGSH iron sulfur domain 1 | HGNC:30880 | 27510639 |
| ACO1 | Aconitase 1 | HGNC:117 | 27514700 |
| ATG13 | Autophagy related 13 | HGNC:29091 | 27514700 |
| ATG16L1 | Autophagy related 16 like 1 | HGNC:21498 | 27514700 |
| ATG3 | Autophagy related 3 | HGNC:20962 | 27514700 |
| ATG4D | Autophagy related 4D cysteine peptidase | HGNC:20789 | 27514700 |
| BECN1 | Beclin 1 | HGNC:1034 | 27514700 |
| G6PDX | _NA_ | _NA_ | 27514700 |
| GABARAPL1 | GABA type A receptor associated protein like 1 | HGNC:4068 | 27514700 |
| GABARAPL2 | GABA type A receptor associated protein like 2 | HGNC:13291 | 27514700 |
| MAP1LC3A | Microtubule associated protein 1 light chain 3 alpha | HGNC:6838 | 27514700 |
| SNX4 | Sorting nexin 4 | HGNC:11175 | 27514700 |
| ULK1 | Unc-51 like autophagy activating kinase 1 | HGNC:12558 | 27514700 |
| ULK2 | Unc-51 like autophagy activating kinase 2 | HGNC:13480 | 27514700 |
| WIPI1 | WD repeat domain, phosphoinositide interacting 1 | HGNC:25471 | 27514700 |
| WIPI2 | WD repeat domain, phosphoinositide interacting 2 | HGNC:32225 | 27514700 |
| SAT1 | Spermidine/spermine N1-acetyltransferase 1 | HGNC:10540 | 27698118 |
| FANCD2 | FA complementation group D2 | HGNC:3585 | 27773819 |
| FTMT | Ferritin mitochondrial | HGNC:17345 | 28066232 |
| ATF4 | Activating transcription factor 4 | HGNC:786 | 28130223 |
| HSPA5 | Heat shock protein family A (Hsp70) member 5 | HGNC:5238 | 28130223 |
| EGFR | Epidermal growth factor receptor | HGNC:3236 | 28297659 |
| MAPK1 | Mitogen-activated protein kinase 1 | HGNC:6871 | 28297659 |
| MAPK3 | Mitogen-activated protein kinase 3 | HGNC:6877 | 28297659 |
| BID | BH3 interacting domain death agonist | HGNC:1050 | 28384611 |
| ZEB1 | Zinc finger E-box binding homeobox 1 | HGNC:11642 | 28678785 |
| DPP4 | Dipeptidyl peptidase 4 | HGNC:3009 | 28813679 |
| FADS2 | Fatty acid desaturase 2 | HGNC:3575 | 28900510 |
| HELLS | Helicase, lymphoid specific | HGNC:4861 | 28900510 |
| SCD | Stearoyl-CoA desaturase | HGNC:10571 | 28900510 |
| SRC | SRC proto-oncogene, non-receptor tyrosine kinase | HGNC:11283 | 28972104 |
| STAT3 | Signal transducer and activator of transcription 3 | HGNC:11364 | 28972104 |
| CDKN2A | Cyclin dependent kinase inhibitor 2A | HGNC:1787 | 28985506 |
| PEBP1 | Phosphatidylethanolamine binding protein 1 | HGNC:8630 | 29053969 |
| PML | Promyelocytic leukemia | HGNC:9113 | 29081404 |
| SOCS1 | Suppressor of cytokine signaling 1 | HGNC:19383 | 29081404 |
| MTOR | Mechanistic target of rapamycin kinase | HGNC:3942 | 29127238 |
| CDO1 | Cysteine dioxygenase type 1 | HGNC:1795 | 29144989 |
| MYB | MYB proto-oncogene, transcription factor | HGNC:7545 | 29144989 |
| NFS1 | NFS1 cysteine desulfurase | HGNC:15910 | 29168506 |
| TP63 | Tumor protein p63 | HGNC:15979 | 29212036 |
| MAPK8 | Mitogen-activated protein kinase 8 | HGNC:6881 | 29330409 |
| MAPK9 | Mitogen-activated protein kinase 9 | HGNC:6886 | 29330409 |
| CDKN1A | Cyclin dependent kinase inhibitor 1A | HGNC:1784 | 29346757 |
| MIR137 | microRNA 137 | HGNC:31523 | 29348676 |
| CHAC1 | ChaC glutathione specific gamma-glutamylcyclotransferase 1 | HGNC:28680 | 29383104 |
| MAPK14 | Mitogen-activated protein kinase 14 | HGNC:6876 | 29436589 |
| ENPP2 | Ectonucleotide pyrophosphatase/phosphodiesterase 2 | HGNC:3357 | 29551679 |
| LINC00472 | Long intergenic non-protein coding RNA 472 | HGNC:21380 | 29588351 |
| FH | Fumarate hydratase | HGNC:3700 | 29917289 |
| CISD2 | CDGSH iron sulfur domain 2 | HGNC:24212 | 29928961 |
| MIR9-1 | microRNA 9-1 | HGNC:31641 | 30035324 |
| MIR9-2 | microRNA 9-2 | HGNC:31642 | 30035324 |
| MIR9-3 | microRNA 9-3 | HGNC:31646 | 30035324 |
| PRKAA1 | Protein kinase AMP-activated catalytic subunit alpha 1 | HGNC:9376 | 30057310 |
| PRKAA2 | Protein kinase AMP-activated catalytic subunit alpha 2 | HGNC:9377 | 30057310 |
| ELAVL1 | ELAV like RNA binding protein 1 | HGNC:3312 | 30081711 |
| BAP1 | BRCA1 associated protein 1 | HGNC:950 | 30202049 |
| CBS | Cystathionine beta-synthase | HGNC:1550 | 30258181 |
| ISCU | Iron-sulfur cluster assembly enzyme | HGNC:29882 | 30557609 |
| ACSL3 | Acyl-CoA synthetase long chain family member 3 | HGNC:3570 | 30686757 |
| CD44 | CD44 molecule (Indian blood group) | HGNC:1681 | 30709928 |
| OTUB1 | OTU deubiquitinase, ubiquitin aldehyde binding 1 | HGNC:23077 | 30709928 |
| ABCC1 | ATP binding cassette subfamily C member 1 | HGNC:51 | 30726737 |
| LINC00336 | Long intergenic non-protein coding RNA 336 | HGNC:33813 | 30787392 |
| MIR6852 | microRNA 6852 | HGNC:49993 | 30787392 |
| ACVR1B | Activin A receptor type 1B | HGNC:172 | 30804470 |
| TGFBR1 | Transforming growth factor beta receptor 1 | HGNC:11772 | 30804470 |
| EPAS1 | Endothelial PAS domain protein 1 | HGNC:3374 | 30962421 |
| HIF1A | Hypoxia inducible factor 1 subunit alpha | HGNC:4910 | 30962421 |
| HILPDA | Hypoxia inducible lipid droplet associated | HGNC:28859 | 30962421 |
| BRD4 | Bromodomain containing 4 | HGNC:13575 | 30988278 |
| PRDX6 | Peroxiredoxin 6 | HGNC:16753 | 31036877 |
| IFNG | Interferon gamma | HGNC:5438 | 31043744 |
| ANO6 | Anoctamin 6 | HGNC:25240 | 31060306 |
| LPIN1 | Lipin 1 | HGNC:13345 | 31061954 |
| HMGB1 | High mobility group box 1 | HGNC:4983 | 31105999 |
| MIR17 | microRNA 17 | HGNC:31547 | 31160087 |
| TNFAIP3 | TNF alpha induced protein 3 | HGNC:11896 | 31160087 |
| TLR4 | Toll like receptor 4 | HGNC:11850 | 31196626 |
| ATF3 | Activating transcription factor 3 | HGNC:785 | 31273299 |
| ATM | ATM serine/threonine kinase | HGNC:795 | 31320750 |
| SESN2 | Sestrin 2 | HGNC:20746 | 31323261 |
| NF2 | Neurofibromin 2 | HGNC:7773 | 31341276 |
| YY1AP1 | YY1 associated protein 1 | HGNC:30935 | 31341276 |
| ARNTL | Aryl hydrocarbon receptor nuclear translocator like | HGNC:701 | 31355331 |
| EGLN2 | Egl-9 family hypoxia inducible factor 2 | HGNC:14660 | 31355331 |
| JUN | Jun proto-oncogene, AP-1 transcription factor subunit | HGNC:6204 | 31394193 |
| MIOX | Myo-inositol oxygenase | HGNC:14522 | 31437128 |
| CA9 | Carbonic anhydrase 9 | HGNC:1383 | 31442913 |
| TAZ | Tafazzin | HGNC:11577 | 31484063 |
| TMBIM4 | Transmembrane BAX inhibitor motif containing 4 | HGNC:24257 | 31507082 |
| PLIN2 | Perilipin 2 | HGNC:248 | 31520166 |
| MTDH | Metadherin | HGNC:29608 | 31527591 |
| MIR212 | microRNA 212 | HGNC:31589 | 31533781 |
| Fer1HCH | Ferritin 1 Heavy Chain Homolog | _NA_ | 31568497 |
| IDH1 | Isocitrate dehydrogenase (NADP(+)) 1 | HGNC:5382 | 31591388 |
| SIRT1 | Sirtuin 1 | HGNC:14929 | 31610175 |
| AIFM2 | Apoptosis inducing factor mitochondria associated 2 | HGNC:21411 | 31634899 |
| LAMP2 | Lysosomal associated membrane protein 2 | HGNC:6501 | 31672277 |
| FBXW7 | F-box and WD repeat domain containing 7 | HGNC:16712 | 31679460 |
| ZFP36 | ZFP36 ring finger protein | HGNC:12862 | 31679460 |
| PANX1 | Pannexin 1 | HGNC:8599 | 31694915 |
| DNAJB6 | DnaJ heat shock protein family (Hsp40) member B6 | HGNC:14888 | 31701262 |
| PROM2 | Prominin 2 | HGNC:20685 | 31735663 |
| BACH1 | BTB domain and CNC homolog 1 | HGNC:935 | 31740582 |
| CHMP5 | Charged multivesicular body protein 5 | HGNC:26942 | 31761326 |
| CHMP6 | Charged multivesicular body protein 6 | HGNC:25675 | 31761326 |
| LONP1 | Lon peptidase 1, mitochondrial | HGNC:9479 | 31822343 |
| CAV1 | Caveolin 1 | HGNC:1527 | 31877357 |
| GCH1 | GTP cyclohydrolase 1 | HGNC:4193 | 31989025 |
| YWHAE | Tyrosine 3-monooxygenase/tryptophan 5-monooxygenase activation protein epsilon | HGNC:12851 | 31581313 |
| MAP3K5 | Mitogen-activated protein kinase kinase kinase 5 | HGNC:6857 | 28887319 |
| LURAP1L | Leucine rich adaptor protein 1 like | HGNC:31452 | 24844246 |
| EIF2S1 | Eukaryotic translation initiation factor 2 subunit 1 | HGNC:3265 | 24844246 |
| EIF2AK4 | Eukaryotic translation initiation factor 2 alpha kinase 4 | HGNC:19687 | 29383104 |
| SLC2A1 | Solute carrier family 2 member 1 | HGNC:11005 | 28900510 |
| SLC2A12 | Solute carrier family 2 member 12 | HGNC:18067 | 28900513 |
| SLC2A14 | Solute carrier family 2 member 14 | HGNC:18301 | 28900515 |
| SLC2A3 | Solute carrier family 2 member 3 | HGNC:11007 | 28900510 |
| SLC2A6 | Solute carrier family 2 member 6 | HGNC:11011 | 28900511 |
| SLC2A8 | Solute carrier family 2 member 8 | HGNC:13812 | 28900512 |
| IL33 | Interleukin 33 | HGNC:16028 | 27352622 |
| IL6 | Interleukin 6 | HGNC:6018 | 25385600 |
| AURKA | Aurora kinase A | HGNC:11393 | 31740746 |
| CAPG | Capping actin protein, gelsolin like | HGNC:1474 | 31108460 |
| DRD4 | Dopamine receptor D4 | HGNC:3025 | 27793671 |
| GABPB1 | GA binding protein transcription factor subunit beta 1 | HGNC:4074 | 31700067 |
| HAMP | Hepcidin antimicrobial peptide | HGNC:15598 | 27773819 |
| HNF4A | Hepatocyte nuclear factor 4 alpha | HGNC:5024 | 31108460 |
| MAFG | MAF bZIP transcription factor G | HGNC:6781 | 26403645 |
| NGB | Neuroglobin | HGNC:14077 | 31405213 |
| RGS4 | Regulator of G protein signaling 4 | HGNC:10000 | 24844246 |
| RRM2 | Ribonucleotide reductase regulatory subunit M2 | HGNC:10452 | 31108460 |
| SNORA16A | Small nucleolar RNA, H/ACA box 16A | HGNC:32605 | 24844246 |
| SP1 | Sp1 transcription factor | HGNC:11205 | 31056284 |
| STEAP3 | STEAP3 metalloreductase | HGNC:24592 | 27773819 |
| STMN1 | Stathmin 1 | HGNC:6510 | 31108460 |
| TFAP2C | Transcription factor AP-2 gamma | HGNC:11744 | 31056284 |
| AGPAT3 | 1-acylglycerol-3-phosphate O-acyltransferase 3 | HGNC:326 | 25965523 |
| ALB | Albumin | HGNC:399 | 24439385 |
| ANGPTL7 | Angiopoietin like 7 | HGNC:24078 | 24439385 |
| ARRDC3 | Arrestin domain containing 3 | HGNC:29263 | 24844246 |
| ASNS | Asparagine synthetase (glutamine-hydrolyzing) | HGNC:753 | 24844246 |
| ATP6V1G2 | ATPase H+ transporting V1 subunit G2 | HGNC:862 | 24844246 |
| BNIP3 | BCL2 interacting protein 3 | HGNC:1084 | 24439385 |
| CEBPG | CCAAT enhancer binding protein gamma | HGNC:1837 | 24844246 |
| CXCL2 | C-X-C motif chemokine ligand 2 | HGNC:4603 | 25385600 |
| DDIT3 | DNA damage inducible transcript 3 | HGNC:2726 | 24844246 |
| DDIT4 | DNA damage inducible transcript 4 | HGNC:24944 | 24844246 |
| DRD5 | Dopamine receptor D5 | HGNC:3026 | 27793671 |
| DUSP1 | Dual specificity phosphatase 1 | HGNC:3064 | 24439385 |
| FTL | Ferritin light chain | HGNC:3999 | 26097885 |
| GDF15 | Growth differentiation factor 15 | HGNC:30142 | 24844246 |
| GPT2 | Glutamic--pyruvic transaminase 2 | HGNC:18062 | 24844246 |
| GPX2 | Glutathione peroxidase 2 | HGNC:4554 | 24439385 |
| HBA1 | Hemoglobin subunit alpha 1 | HGNC:4823 | 31108460 |
| HERPUD1 | Homocysteine inducible ER protein with ubiquitin like domain 1 | HGNC:13744 | 24844246 |
| HIC1 | HIC ZBTB transcriptional repressor 1 | HGNC:4909 | 31108460 |
| HSD17B11 | Hydroxysteroid 17-beta dehydrogenase 11 | HGNC:22960 | 25965523 |
| JDP2 | Jun dimerization protein 2 | HGNC:17546 | 24844246 |
| KLHL24 | Kelch like family member 24 | HGNC:25947 | 24844246 |
| MT3 | Metallothionein 3 | HGNC:7408 | 24439385 |
| NCF2 | Neutrophil cytosolic factor 2 | HGNC:7661 | 24439385 |
| NNMT | Nicotinamide N-methyltransferase | HGNC:7861 | 31108460 |
| NOS2 | Nitric oxide synthase 2 | HGNC:7873 | 24439385 |
| OXSR1 | Oxidative stress responsive kinase 1 | HGNC:8508 | 24439385 |
| PCK2 | Phosphoenolpyruvate carboxykinase 2, mitochondrial | HGNC:8725 | 24844246 |
| PLIN4 | Perilipin 4 | HGNC:29393 | 31108460 |
| PRDX1 | Peroxiredoxin 1 | HGNC:9352 | 31901729 |
| PSAT1 | Phosphoserine aminotransferase 1 | HGNC:19129 | 24844246 |
| PTGS2 | Prostaglandin-endoperoxide synthase 2 | HGNC:9605 | 24439385 |
| RIPK1 | Receptor interacting serine/threonine kinase 1 | HGNC:10019 | 31827280 |
| SETD1B | SET domain containing 1B, histone lysine methyltransferase | HGNC:29187 | 25965523 |
| SLC1A4 | Solute carrier family 1 member 4 | HGNC:10942 | 24844246 |
| SLC7A5 | Solute carrier family 7 member 5 | HGNC:11063 | 24844246 |
| SRXN1 | Sulfiredoxin 1 | HGNC:16132 | 24439385 |
| TRIB3 | Tribbles pseudokinase 3 | HGNC:16228 | 24844246 |
| TSC22D3 | TSC22 domain family member 3 | HGNC:3051 | 24844246 |
| TUBE1 | Tubulin epsilon 1 | HGNC:20775 | 24844246 |
| TXNIP | Thioredoxin interacting protein | HGNC:16952 | 24844246 |
| TXNRD1 | Thioredoxin reductase 1 | HGNC:12437 | 24439385 |
| UBC | Ubiquitin C | HGNC:12468 | 24439385 |
| VEGFA | Vascular endothelial growth factor A | HGNC:12680 | 24844246 |
| VLDLR | Very low density lipoprotein receptor | HGNC:12698 | 24844246 |
| XBP1 | X-box binding protein 1 | HGNC:12801 | 24844246 |
| ZNF419 | Zinc finger protein 419 | HGNC:20648 | 24844246 |
| MIR30B | microRNA 30b | HGNC:31625 | 31926626 |
| MIR4715 | microRNA 4715 | HGNC:41666 | 31740746 |
| BLOC1S5-TXNDC5 | BLOC1S5-TXNDC5 readthrough (NMD candidate) | HGNC:42001 | 24844246 |
| GLUT13 | _NA_ | _NA_ | 28900514 |
| LOC390705 | _NA_ | _NA_ | 24844246 |
| KIM-1 | Kidney injury molecule-1 | _NA_ | 25385600 |
| LOC284561 | _NA_ | _NA_ | 24844246 |
| RELA | RELA proto-oncogene, NF-kB subunit | HGNC:9955 | 25385600 |
| SELENOS | Selenoprotein S | HGNC:30396 | 24439385 |
| ZFP69B | ZFP69 zinc finger protein B | HGNC:28053 | 24844246 |
