# Supplementary material for: Identification of ferroptosis‐related genes as potential biomarkers of tongue squamous cell carcinoma using an integrated bioinformatics approach
Source: FEBS Open Bio. 2021 Dec 24;12(2):412–29. doi: 10.1002/2211-5463.13348 (PMC8804613; doi:10.1002/2211-5463.13348)
Supplement: Supplementary file 2 — Table S2. Expression information of CA9, TNFAIP3, and NRAS. [file FEB4-12-412-s002.docx]

Supplementary Table 2 Expression information of CA9, TNFAIP3, and NRAS

| **Sample ID** | **CA9** | **TNFAIP3** | **NRAS** |
| --- | --- | --- | --- |
| TCGA-BB-4224-01A | high | low | low |
| TCGA-CV-6943-01A | high | high | high |
| TCGA-CV-6959-01A | high | high | high |
| TCGA-F7-A61V-01A | high | low | low |
| TCGA-CV-A45T-01A | low | low | low |
| TCGA-DQ-7592-01A | high | high | high |
| TCGA-BA-4075-01A | high | high | high |
| TCGA-MZ-A7D7-01A | low | low | high |
| TCGA-QK-A652-01A | low | low | low |
| TCGA-CV-6433-01A | high | high | low |
| TCGA-CN-6998-01A | high | high | high |
| TCGA-CV-6941-01A | high | high | low |
| TCGA-CQ-5325-01A | low | low | low |
| TCGA-BB-4225-01A | low | high | high |
| TCGA-T2-A6WZ-01A | high | low | low |
| TCGA-HD-8314-01A | low | low | low |
| TCGA-CN-A642-01A | high | low | low |
| TCGA-BB-A6UO-01A | high | low | low |
| TCGA-DQ-7591-01A | low | high | high |
| TCGA-HD-8634-01A | low | low | low |
| TCGA-CR-7372-01A | high | low | low |
| TCGA-HD-8224-01A | low | low | low |
| TCGA-BA-4074-01A | high | low | high |
| TCGA-CR-7392-01A | low | low | low |
| TCGA-CQ-5329-01A | high | low | high |
| TCGA-H7-A6C4-01A | low | high | low |
| TCGA-BA-A6DB-01A | low | low | low |
| TCGA-MT-A51X-01A | low | low | high |
| TCGA-4P-AA8J-01A | low | low | low |
| TCGA-CV-5439-01A | high | high | high |
| TCGA-D6-A4Z9-01A | low | high | high |
| TCGA-CQ-5327-01A | low | low | high |
| TCGA-CX-7085-01A | low | high | high |
| TCGA-F7-A50J-01A | low | high | low |
| TCGA-CQ-6222-01A | high | low | low |
| TCGA-D6-6825-01A | high | high | high |
| TCGA-CV-6939-01A | low | high | high |
| TCGA-CR-6477-01A | high | high | high |
| TCGA-WA-A7H4-01A | low | low | low |
| TCGA-CR-7397-01A | low | low | low |
| TCGA-F7-A61W-01A | low | low | high |
| TCGA-CV-5976-01A | low | high | high |
| TCGA-CV-6954-01A | high | high | high |
| TCGA-CV-6961-01A | high | high | high |
| TCGA-CV-A45R-01A | low | low | low |
| TCGA-CQ-6219-01A | high | low | high |
| TCGA-P3-A5QE-01A | low | high | low |
| TCGA-CN-6016-01A | low | low | low |
| TCGA-BA-A6DG-01A | high | high | low |
| TCGA-F7-A50G-01A | low | low | high |
| TCGA-BA-6871-01A | high | high | high |
| TCGA-CR-6488-01A | high | low | high |
| TCGA-CN-5370-01A | high | high | high |
| TCGA-CV-7104-01A | high | high | high |
| TCGA-CV-A45P-01A | low | low | low |
| TCGA-CV-7238-01A | high | low | low |
| TCGA-CV-5971-01A | low | low | low |
| TCGA-IQ-A61H-01A | high | low | low |
| TCGA-CV-6956-01A | high | high | high |
| TCGA-CQ-6221-01A | low | low | high |
| TCGA-HD-8635-01A | low | low | low |
| TCGA-CV-6934-01A | high | high | high |
| TCGA-CV-7406-01A | low | high | high |
| TCGA-CQ-5333-01A | high | low | low |
| TCGA-CN-4737-01A | high | low | low |
| TCGA-C9-A480-01A | low | low | low |
| TCGA-CV-6003-01A | high | high | high |
| TCGA-F7-A61S-01A | high | low | low |
| TCGA-CV-6933-01A | high | high | high |
| TCGA-DQ-5630-01A | low | high | high |
| TCGA-UP-A6WW-01A | high | low | low |
| TCGA-IQ-A61J-01A | low | low | low |
| TCGA-CN-4742-01A | low | high | high |
| TCGA-BA-A6DE-01A | low | high | low |
| TCGA-CN-6017-01A | low | high | low |
| TCGA-CV-A6JU-01A | low | low | low |
| TCGA-CR-7401-01A | low | low | low |
| TCGA-CR-6493-01A | high | high | high |
| TCGA-CV-6436-01A | high | low | low |
| TCGA-DQ-5625-01A | high | high | high |
| TCGA-QK-AA3K-01A | high | low | low |
| TCGA-HD-7831-01A | low | high | low |
| TCGA-CR-5250-01A | low | high | high |
| TCGA-CN-A498-01A | high | high | high |
| TCGA-CV-7103-01A | high | low | low |
| TCGA-DQ-5631-01A | high | high | high |
| TCGA-BA-6873-01A | high | low | high |
| TCGA-CQ-6229-01A | high | low | high |
| TCGA-CV-6441-01A | low | low | high |
| TCGA-CV-6952-01A | high | high | high |
| TCGA-CN-A6V6-01A | low | low | low |
| TCGA-CN-6019-01A | low | high | low |
| TCGA-CQ-5330-01A | low | low | low |
| TCGA-DQ-5624-01A | low | high | high |
| TCGA-CQ-A4CE-01A | low | low | low |
| TCGA-CV-5973-01A | high | high | low |
| TCGA-CV-A6JT-01A | high | low | low |
| TCGA-CV-7446-01A | low | high | high |
| TCGA-CN-A6UY-01A | low | high | low |
| TCGA-CV-A465-01A | low | low | high |
| TCGA-D6-A4ZB-01A | low | high | low |
| TCGA-CV-7438-01A | high | high | high |
| TCGA-CV-7180-01A | low | low | low |
| TCGA-CV-6950-01A | high | high | high |
| TCGA-CQ-A4CH-01A | low | high | high |
| TCGA-CR-7394-01A | low | low | low |
| TCGA-CQ-6218-01A | high | high | high |
| TCGA-CQ-7072-01A | low | low | low |
| TCGA-CN-4725-01A | high | high | high |
| TCGA-BB-4228-01A | high | high | high |
| TCGA-CV-5970-01A | high | high | high |
| TCGA-CQ-7065-01A | low | low | high |
| TCGA-CR-7382-01A | low | high | high |
| TCGA-CQ-6224-01A | high | low | high |
| TCGA-D6-6823-01A | high | high | high |
| TCGA-CV-6945-01A | high | low | low |
| TCGA-CR-7393-01A | low | high | low |
| TCGA-MT-A67A-01A | low | low | low |
| TCGA-IQ-A6SG-01A | high | high | high |
| TCGA-CV-5977-01A | high | low | low |
| TCGA-CR-7391-01A | low | low | low |
| TCGA-F7-A620-01A | low | high | low |
| TCGA-KU-A6H8-01A | high | low | low |
| TCGA-CN-5367-01A | high | low | high |
| TCGA-D6-6515-01A | low | low | high |
| TCGA-D6-8569-01A | low | low | low |
| TCGA-BA-7269-01A | low | low | low |
| TCGA-UF-A7JS-01A | low | low | low |
| TCGA-CN-6996-01A | high | high | high |
| TCGA-IQ-A61E-01A | high | high | high |
| TCGA-P3-A5QA-01A | high | high | high |
| TCGA-CR-6472-01A | low | low | high |
| TCGA-CN-6024-01A | high | high | low |
| TCGA-CV-7255-01A | high | high | high |
| TCGA-CN-4736-01A | low | high | high |
| TCGA-CV-7236-01A | low | high | high |
| TCGA-CV-6951-01A | low | low | low |
| TCGA-CV-5979-01A | high | high | high |
| TCGA-CN-4733-01A | low | high | low |
| TCGA-CR-7390-01A | high | high | high |
| TCGA-IQ-A6SH-01A | low | low | low |
| TCGA-C9-A47Z-01A | high | high | low |
| TCGA-HD-A6HZ-01A | high | high | low |
